# 3-month prevalence of unwanted sexual contact victimization in a national sample of college students: differences by race, gender identity, and sexual identity

**DOI:** 10.1186/s12889-024-18018-7

**Published:** 2024-02-22

**Authors:** Lisa Fedina, Anna E. Bender, Meggie Royer, Louise Ashwell, Richard Tolman, Todd I. Herrenkohl

**Affiliations:** 1 School of Social Work, University of Michigan, 1080 S. University Ave, 48109 Ann Arbor, MI USA; 2 Harborview Injury Prevention & Research Center, University of Washington, Washington, USA

**Keywords:** Campus sexual assault, Sexual violence, Dating violence, Harassment, LGBTQ+, Race

## Abstract

**Importance:**

Most unwanted sexual contact victimization (USCV) research utilizes predominantly white, cisgender, heterosexual college student samples. Estimates of USCV prevalence and demographic variation can determine the need for dedicated funding and culturally relevant campus services for students in high-risk groups.

**Objective:**

To estimate the national prevalence and demographic variation in self-reported USCV within the first three months of college.

**Design:**

Data are from the *Sexual Assault Prevention for Undergrads (SAPU)* (2020–2021) dataset. SAPU is an online intervention program administered to students on more than 600 college campuses in the United States (*N =* 250,359). Group differences were assessed by race/ethnicity, gender identity, and sexual identity, and then stratified by gender to assess within-gender group differences.

**Setting:**

The SAPU dataset includes public and private institutions and 2-year and 4-year colleges with varying sizes of enrollment.

**Participants:**

The sample is demographically diverse, and consists of newly matriculated U.S. college students, most of whom complete the SAPU program within the first three months of enrollment.

**Main outcomes and measures:**

The primary outcome measure is self-reported USCV within the first three months of college enrollment, analyzed for subgroup differences. We hypothesized that USCV would be higher among students from racial/ethnic, gender, and sexual minority populations.

**Results:**

Nearly 8% of transgender men reported USCV, followed by 7.4% of transgender women, 7.4% of genderqueer/gender non-conforming students, 4.5% of women, and 1.5% of men. Several subgroups reported exceedingly high rates of USCV, including Black students who identified as transgender women (35.7%) and American Indian/Alaska Native/Native Hawaiian/Pacific Islander students who identified as trans men (55.6%) or genderqueer/gender non-conforming (41.7%).

**Conclusions and relevance:**

Universal and targeted (selective and indicated) intervention programs are needed to lessen USCV, particularly among gender minority students who also identify as Black, Indigenous, other person of color, or as a sexual minority.

## Introduction

Preventing sexual assault among college students in the United States is a national priority [1]. Prevalence studies have found that 1 in 4 or 1 in 5 undergraduate cisgender women are sexually assaulted while attending college [2]. The few studies that have examined campus sexual assault among subgroups find that sexual and gender minorities and some populations of students of color are at higher risk for sexual assault victimization than students of dominant majority groups (i.e., heterosexual, cisgender, white) [3, 4]. The 2020 report from the Association of American Universities (AAU) Campus Climate Survey on Sexual Assault and Sexual Misconduct found that bisexual students reported the highest rate of unwanted sexual contact victimization (25.6%), followed by students identifying with more than one sexual orientation category (22.2%), asexual/queer/questioning or other sexual orientation students (18.5%), and gay or lesbian students (15.1%) compared to 11.5% of heterosexual students [5]. This same study found that cisgender women and gender minority students (i.e., transgender women, transgender men, nonbinary or genderqueer, gender questioning or gender not listed) experienced sexual victimization at higher rates compared to cisgender men students [5]. Research has found fewer significant differences in rates of victimization across race ethnicity, with some patterns suggesting higher rates among Latino students and lower victimization rates among Asian students [5] However, cisgender women of color and SGM students of color may experience higher rates of victimization than other racial/ethnic student populations within gender and sexual orientation subgroups [3, 4]. Relatedly, the only review to date on sexual victimization experiences among SGM students of color indicates that, not only do these students experience greater victimization risk than heterosexual, cisgender, white students, but they also experience more types of victimization overall [3], including intimate partner violence victimization.

Intersectional research on campus sexual assault among sexual and gender minority (SGM) students of color is even more limited. In one of the few studies assessing the intersection of race/ethnicity and SGM identities, Coulter et al [6] found that bisexual cisgender female and bisexual cisgender male students were at higher risk for sexual assault than cisgender heterosexual students, and gay cisgender male students were at higher risk for sexual assault than heterosexual men. In this same study, Black transgender students were at higher risk for sexual assault than white transgender students [6]. The convergence of both interpersonal and structural discrimination can amplify victimization risks for SGM students of color.

Elevated victimization risk among sexual minority, gender minority, and students of color may be due to structural, social, and economic inequities, including barriers to accessing culturally relevant services and lack of resources on college campuses [4–8]. Data on the prevalence of unwanted sexual contact and potential demographic variation in exposure is critical for developing universal, selective, and indicated prevention strategies, and for tailoring interventions to specific subgroups of students who are differentially affected by unwanted sexual contact.

We analyzed data from the 2020–2021 *Sexual Assault Prevention for Undergraduates (SAPU)* dataset [9]. SAPU is a population-level, digital campus sexual assault intervention program delivered to college students in all 50 U.S. states. The large, demographically diverse sample allowed for analyses of subgroup differences that generally are too small to analyze independently. We present a descriptive portrait of unwanted sexual contact victimization (USCV) among student racial/ethnic, gender identity, and sexual identity subpopulations, as well as racial/ethnic differences within SGM identities. We discuss the public health implications of these findings and highlight areas for future study of USCV on college campuses to guide policy and prevention strategies on college campuses.

## Methods

SAPU data consist of pre-intervention and post-intervention data gathered from U.S. newly matriculated college students [10], including public, private, and 2- and 4-year institutions. Pre- and post-tests are typically administered within the first three months of enrollment (mean duration between pre-post tests: *M* = 55.94 days; *SD* = 34.75). Self-reports of USCV that occurred before arriving to campus are also gathered at both time points, which allowed us to account for victimization that occurred prior to college entry (e.g., during childhood or adolescence). Students with data on both pre- and post-test measures were included in the analysis (*N* = 250,359). See Fedina et al. [9] for additional details on study methods and measures. Additionally, the purpose of this study was not to evaluate the effects of the SAPU intervention. Although it is possible that intervention effects influenced rates of USCV, it is unlikely to have had differential effects across student subgroups. See Zapp et al [10] for additional details on the SAPU intervention and its effects on student outcomes. The current study used anonymized data which was determined as exempt research by the University of Michigan Institutional Review Board.

### SAPU survey measures

USCV was assessed with the following item on both the pre- and post-test: “*Has someone ever had unwanted sexual contact with you (e.g., used physical force or threatened to physically harm you; manipulated you through lies, threats, or pressure; took sexual advantage of you when you were significantly impaired or incapacitated by drugs/alcohol, etc.)?* Original response options included “never,” “before college,” “since college,” “both before and during college,” “not sure,” or “prefer not to answer.” Responses were recoded into three categories: 1) never, 2) before college only, or 3) any exposure during college (i.e., at the time of pre-post test survey administration which was approximately the first 3 months of enrollment). In the original response options, students who endorsed USCV “during college” or “both before and during college” on either the pre- or post-test were categorized as experiencing USCV during college to capture all reports of USCV since entering college. Students who provided non-definitive answers (i.e., “not sure,” “preferred not to answer”) were removed from the analysis. A positive endorsement of USCV on either the pre- or post-test measure was coded as a USCV exposure (before college only or any exposure to college). Participants who indicated they never experienced USCV on both the pre- and post-test were coded as having no exposure (never).

Gender identity was assessed with the question *“What is your current gender identity?*” which included eight original response options: “woman,” “man,” “transgender woman,” “transgender man,” “genderqueer,” “gender-nonconforming,” “other gender identity not listed,” and “prefer not to answer.” Gender identity was recoded into six categories: “woman,” “man,” transgender woman, transgender man, genderqueer/gender-nonconforming/other gender identity, and prefer not to answer. Participants identifying as genderqueer, gender-nonconforming, or other gender identity not listed were combined due to small within-group sample sizes.

Sexual identity was assessed with the question *“How do you self-identify?”* and had 10 original response options with the choice to select more than one sexual identity: “bisexual,”

“gay,” “heterosexual/straight,” “lesbian,” “queer,” “questioning,” “pansexual,” “asexual,” “other.

sexual orientation not listed,” and “prefer not to answer.” Participants who selected more than one sexual identity were recoded as “multiple sexual identities,” and participants who identified as queer, pansexual, questioning, or other sexual orientation not listed were combined due to small within-group sample sizes.

Racial and ethnic identity was assessed with the question *“Select one or more of the*.

*following options that best describes your race?”* This question included eight original response options and the ability to select more than one race: “American Indian or Alaska Native,” “Asian,” “Black or African American,” “Hispanic or Latino/a,” “Middle Eastern or North African,” “Native Hawaiian or Other Pacific Islander,” “white/Caucasian,” and “other race not listed.” Race was recoded into six mutually exclusive categories, and participants who identified with more than one racial/ethnic identity were recoded as multiracial. Participants who identified as American Indian/Alaska Native (AIAN) and Native Hawaiian/Pacific Islander (NHPI) were.

combined due to small sample sizes. Due to very small frequencies (*n* = 65 or 0% of the total sample), participants who identified as Middle Eastern or North African (only) or as other race not listed (only) were not included in the analysis.

### Statistical analyses

Descriptive and bivariate statistics were examined to determine the overall prevalence of USCV by race/ethnicity, gender identity, and sexual identity. The sample was then stratified by gender identity and sexual identity to examine within-group differences by race/ethnicity to compare results with prior studies [2, 4]. We conducted sensitivity analyses for participants who completed only the pre-test (*n* = 405,217) and participants who completed both the pre- and post-test (*n* = 250,359). Demographic differences in USCV were consistent for these samples. Results for participants who completed both the pre- and post-test are presented below (*N* = 250,359). All analyses were conducted using IMB SPSS Statistics (Version 29).

## Results

**Fig. 1:**
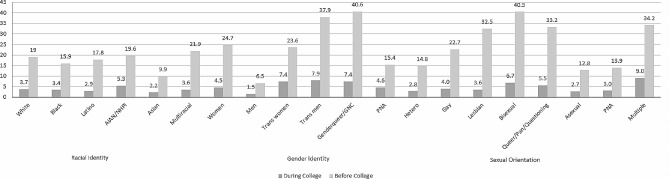
Prevalence rate of self-reported USCV before college and during college (within approximately first 3 months) across racial/ethnic, gender identity, and sexual identity subpopulations (N = 250,359). Significant differences noted between groups for race/ethnicity (X2 = 1874.49, df = 10; *p* <.001; phi =.091), gender identity (X2 = 15919.83, df = 10; *p* <.001; phi =.262), and sexual identities (X2 = 10580.92, df = 14; *p* <.001; phi =.22). Notes. AIAN/NHPI = American Indian or Alaska Native/Native Hawaiian or Pacific Islander. GNC = gender non-conforming. PNA = prefer not to answer. Multiple sexual identities = two or more sexual identities

Rates of USCV before and during college (within approximately the first 3 months) are described below and presented in Fig. 1; Table 1 by race/ethnicity, gender identity, and sexual identity. Descriptive statistics and effect sizes for racial/ethnic differences in USCV within gender identity and sexual identity groups are presented in Tables 2 and 3. A large proportion of SGM students reported USCV before entering college. Over a third of transgender men (37.9%), gender queer (40.6%) students, and bisexual students (40.5%) reported USCV before entering college. With regard to race/ethnicity, 1 in 4 multiracial students (21.9%) reported USCV before entering college, followed by 19.6% of AIAN/NHPI students, 19% of white students, 17.8% of Latino students, 15.9% of Black/African American students, and 9.9% of Asian students.

**Table 1:** Unwanted Sexual Contact Victimization (USCV) Before College and During (First 3 Months) of College Within Gender Identity, Sexual Identity, and Racial/Ethnic Groups (*N* = 250,359)

Demographic	USCV	USCV
	Before College	(First 3 Months)
	% (n)	% (n)
Gender Identity***		
Woman	24.7% (33,812)	4.5% (6194)
Man	6.5% (5887)	1.5% (1381)
Transgender Woman	23.6% (61)	7.4% (19)
Transgender Man	37.9% (230)	7.9% (48)
Genderqueer/Gender non-conforming/Other Gender	40.6% (938)	7.4% (172)
PNA	15.4% (311)	4.6% (93)
Sexual Identity***		
Heterosexual	14.8% (24,879)	2.8% (4775)
Gay	22.7% (741)	4.0% (130)
Lesbian	32.5% (815)	3.6% (91)
Bisexual	40.5% (6569)	6.7% (1091)
Queer/Pansexual/Questioning/Other	33.2% (2547)	5.5% (422)
Asexual	12.8% (872)	2.7% (185)
PNA	13.9% (1013)	3.0% (219)
Multiple sexual identities	34.2% (628)	9.0% (166)
Race/Ethnicity***		
White	19.0% (25,442)	3.7% (4954)
Black/African American	15.9% (2882)	3.4% (617)
Latino	17.8% (4335)	2.9% (712)
AIAN/NHPI	19.6% (227)	5.3% (61)
Asian	9.9% (2668)	2.2% (579)
Multiracial	21.9% (5209)	3.6% (857)

Notes. *** Denotes statistically significant group differences at *p* <.001. Significant differences noted between groups for race/ethnicity (X2 = 1874.49, df = 10; *p* <.001; phi = 0.09), gender identity (X2 = 15919.83, df = 10; *p* <.001; phi = 0.26), and sexual identities (X2 = 10580.92, df = 14; *p* >.001; phi = 0.22). USCV “during college” capture rates within (approximately) the first 3 months of college as pre- and post-tests were administered within the first three months of enrollment. Totals reflect percentage of students within gender and sexual identities and racial groups reporting unwanted sexual contact victimization (USCV). Students who selected two or more sexual identities (e.g., lesbian and queer; asexual and questioning) were coded as having multiple sexual identities. AIAN/NHPI = American Indian or Alaska Native/Native Hawaiian or Pacific Islander. GNC = gender non-conforming. PNA = prefer not to answer

**Table 2:** Racial/Ethnic Differences of Rates of Unwanted Sexual Contact Victimization (USCV) Before College and During (First 3 Months) of College Within Gender Groups (*N* = 228,218)

	Women***		Men***		Transgender Women**		Transgender Men***		Genderqueer/ Gender Non-conforming/Other gender***		Prefer Not to Answer	
**Race**	*% (n)*	*phi*	*% (n)*	*phi*	*% (n)*	*phi*	*% (n)*	*phi*	*% (n)*	*phi*	*% (n)*	*phi*
		0.1		0.6		0.3		0.3		0.2		0.1
White												
Before college	26.5 (20,773)		7 (3711)		23.8 (38)		42.2 (170)		41.8 (573)		16.1 (151)	
During college	5 (3958)		1 0.6 (824)		5 (8)		5.5 (22)		6.3 (87)		5.2 (49)	
Black/African American												
Before college	20.4 (2410)		6 (355)		21.4 (3)		25.9 (7)		44.6 (79)		17.6 (21)	
During college	4.1 (481)		1.7 (100)		35.7 (5)		25.9 (7)		8.5 (15)		5.9 (7)	
Latino												
Before college	24.2 (3699)		6.1 (529)		23.1 (3)		36 (9)		39.4 (65)		22 (27)	
During college	3.7 (566)		1.4 (120)		0 (0)		12 (3)		10.9 (18)		4.1 (5)	
AIAN/NHPI												
Before college	27.7 (187)		6.5 (27)		36.4 (4)		0 (0)		25 (6)		15 (3)	
During college	4.7 (32)		2.6 (11)		18.2 (2)		55.6 (5)		41.7 (10)		5 (1)	
Asian												
Before college	14.9 (2182)		3.4 (401)		16.7 (4)		15.8 (6)		25.5 (41)		14.2 (32)	
During college	2.9 (429)		1.1 (127)		8.3 (2)		2.6 (1)		7.5 (12)		2.7 (6)	
Multiracial												
Before college	30.1 (4199)		8.4 (765)		28.1 (9)		35.2 (32)		44.6 (161)		19.3 (39)	
During college	4.7 (657)		1.7 (158)		6.3 (2)		8.8 (8)		6.6 (24)		3 (6)	

Note. ***Denotes statistically significant group differences at *p* <.001. **Denotes statistically significant group differences a *p* <.01. Phi and chi-square values were obtained for each gender identity group assessing differences by race/ethnicity and sexual identity. Phi values are significant at *p* <.001. USCV “during college” capture rates within (approximately) the first 3 months of college as pre- and post-tests were administered within the first three months of enrollment. Students who selected two or more sexual identities (e.g., lesbian and queer; asexual and questioning) were coded as having multiple sexual identities. AIAN/NHPI = American Indian/Alaska Native or Native Hawaiian/Pacific Islander

**Table 3:** Racial/Ethnic Differences in Rates of Unwanted Sexual Contact Victimization (USCV) Before College and During (First 3 Months) College Within Sexual Identity Groups (*N* = 206,968)

	Heterosexual***		Gay***		Lesbian**		Bisexual***		Queer/Pansexual/		Asexual***		Multiple Sexual Identities^***^		Prefer Not to Answer***	
									Questioning/Other***							
**Race**	*% (n)*	*phi*	*% (n)*	*phi*	*% (n)*	*phi*	*% (n)*	*phi*	*% (n)*	*phi*	*% (n)*	*phi*	*% (n)*	*phi*	*% (n)*	*phi*
		0.1		0.1		0.1		0.1		0.1		0.1		0.1		0.1
White																
Before college	15.8 (15,473)		25.2 (455)		34.5 (511)		43.2 (3923)		34.4 (1416)		13.3 (493)		37.8 (373)		16.5 (503)	
During college	3.1 (3071)		4.0 (72)		3.2 (48)		7.5 (683)		5.8 (237)		2.7 (98)		9.8 (97)		3.1 (95)	
Black/African American																
Before college	13.4 (1635)		20.7 (47)		27.7 (66)		33.0 (362)		32.6 (199)		11.0 (54)		31.6 (55)		13.6 (83)	
During college	2.9 (354)		5.3 (12)		6.7 (16)		6.5 (71)		6.6 (40)		2.0 (10)		5.7 (10)		3.1 (19)	
Latino																
Before college	14.7 (2358)		20.3 (85)		26.7 (60)		40.4 (671)		34.6 (251)		14.3 (85)		34.3 (61)		13.3 (108)	
During college	2.4 (380)		2.9 (12)		3.6 (8)		5.5 (91)		5.1 (37)		1.8 (11)		9.6 (17)		1.4 (11)	
AIAN/NHPI																
Before college	17.4 (126)		16.7 (3)		26.7 (4)		45.1 (37)		15.6 (5)		14.8 (13)		36.4 (4)		9.3 (5)	
During college	2.8 (20)		27.8 (5)		6.7 (1)		12.2 (10)		12.5 (4)		10.2 (9)		18.2 (2)		5.6 (3)	
Asian																
Before college	8.2 (1535)		12.9 (37)		24.1 (34)		25.8 (385)		21.4 (162)		9.0 (96)		18.7 (36)		7.6 (95)	
During college	1.7 (316)		3.5 (10)		2.1 (3)		4.0 (60)		3.3 (25)		3.1 (33)		7.3 (14)		3.1 (39)	
Multiracial																
Before college	17.7 (2807)		22.2 (82)		36.3 (109)		43.9 (901)		37.4 (389)		17.1 (89)		34.2 (93)		19.2 (143)	
During college	3.0 (480)		4.6 (17)		3.0 (9)		6.4 (131)		5.4 (56)		2.3 (12)		8.5 (23)		3.1 (23)	

Note. *** Denotes statistically significant group differences at < 0.001. ** Denotes statistically significant group differences at < 0.01. Phi and chi-square values were obtained for each gender identity group assessing differences by race/ethnicity and sexual identity Phi values are significant at *p* <.001. USCV “during college” capture rates within (approximately) the first 3 months of college as pre- and post-tests were administered within the first three months of enrollment. Students who selected two or more sexual identities (e.g., lesbian and queer; asexual and questioning) were coded as having multiple sexual identities. AIAN/NHPI = American Indian/Alaska Native or Native Hawaiian/Pacific Islander

Significant differences in USCV within the first 3 months of college were found between racial/ethnic, gender, and sexual identity groups (see Table 1). Students who identified as American Indian/Alaska Native or Native Hawaiian/Pacific Islander (AIAN/NHPI; 5.3%) reported the highest rate of USCV compared to other racial groups including multiracial (4.9%), white (3.7%), Black (3.4%), Latino (2.9%), and Asian (2.2%). Transgender men (7.9%) reported the highest rate of USCV among all genders, followed by transgender women (7.4%), genderqueer/non-conforming (7.4%), women (4.5%), and men (1.5%). Students who identified with more than one or multiple sexual identities (e.g., bisexual and queer) reported the highest rate of USCV (9%), followed by students who identified as bisexual (6.7%), queer/pansexual/questioning (5.5%), gay (4%), lesbian (3.6%), heterosexual (2.8%), and asexual (2.7%).

Significant differences by race/ethnicity and sexual identity were found within each gender group and sexual identity group. Among AIAN/NHPI students, 4.7% of women, 2.6% of men, 55.6% of transgender men, and 41.7% of genderqueer/non-conforming students reported USCV within the first 3 months of college. Among transgender women, those who identified as Black (35.7%) reported the highest rate of USCV compared to other racial groups (see Table 2). Among bisexual students, AIAN/NHPI students reported the highest rate of USCV within the first 3 months of college (12.2%), followed by white (7.5%), Black (6.5%), multiracial (6.4%), Latino (5.5%), and Asian (4%) students (see Table 3).

## Discussion

Results provide further evidence that sexual and gender minority students of color, particularly AIAN, NHPI, and Black students, are at higher risk for USCV than are white, cisgender, heterosexual students. Moreover, results highlight elevated victimization risks faced by transgender male and transgender female students, who are often overlooked in studies of campus sexual assault or combined with other gender minority populations, as well as sexual minority populations, due to methodological challenges (e.g., small sample sizes). Studies from previous general population samples document disproportionately higher rates of sexual assault and interpersonal violence for AIAN and multiracial men and women [11, 12]. Our study extends this knowledge to AIAN and NHPI college students, including AIAN and NHPI students from certain gender minority groups.

Further research is needed to disaggregate data and better understand rates of USCV among AIAN and NHPI student subgroups, including Southeast Asian populations, and to consider how experiences of racism intersect with sexual violence victimization [13]. Additionally, further research should focus on the specific experiences of Black sexual and gender minority students and the compounding impacts of anti-Blackness, racial fetishization, transphobia, and homophobia on sexual violence victimization [14, 15]. Scholars have argued that Black LGBTQ + students may be at greater risk for sexual assault due to racist and hypersexualized school climates, which reinforce racist stereotypes around Black student sexualities while further perpetuating homophobia [14]. 

Attention should also focus on identifying mechanisms leading to higher rates of USCV among students of color and LGBTQIA + students, including the role of structural, institutional, and community-level barriers such as service access, legal protections, racial discrimination, immigration status, and stigma [15–17]. There is a need to understand campus characteristics associated with USCV across student identity groups that can serve as potential intervention and prevention targets at the institutional level, including programs, policies, and climate. Little attention has been focused on understanding campus level factors such as institution size, demographic composition, and type of institution that contribute to victimization. This lack of attention limits opportunities for prevention programming [18] and obscures how campus level predictors may differentially impact students across racial/ethnic, gender, and sexual minority identities.

## Limitations

Findings should be interpreted with several limitations in mind. First, single item assessments of USCV were used in SAPU pre- and post-test surveys, which may underestimate the prevalence of unwanted sexual contact. Relatedly, pre- and post-tests were typically collected within the first three months of enrollment and do not capture experiences throughout a student’s time in college. However, prior research indicates that the first three months of college represent a very high-risk period for USCV [2, 19]. Although sample participants represent a very large and demographically diverse sample of college students in all 50 U.S. states, the SAPU program and dataset do not include probability-based sampling methods and thus, generalizability of findings may be affected. Relatedly, despite the large sample size in the SAPU dataset, caution is warranted when interpreting findings for some underrepresented groups (e.g., AIAN/NHPI transgender women reporting USCV during college). Further research is needed to assess the relative risk for USCV among student subpopulations and to determine whether students with prior victimization experiences are more at risk than are others [20]. Finally, gender identity measures were limited in SAPU data and research on identity measures that are in line with affirming practices, such as assessing sex assigned at birth and including the terms “cisgender man” and “cisgender woman” in response options, can facilitate best practices for capturing data among gender and sexual minority student subgroups. Holmes [21] also argues that expansive demographic questions can broaden sample sizes of non-heterosexual and non-cisgender students and facilitate more nuanced prevalence rates.

## Conclusions

Demographic variation in USCV highlights the need for prioritized funding, resources, and culturally relevant campus services for certain student subpopulations. These services and resources are especially needed for AIAN, NHPI, Black students, and other students of color, including multiracial students, who also identify as gender and sexual minorities. Student survivors and scholars have recommended that campus prevention programming address the larger social context of victimization risk and experiences for minority student subgroups, including discrimination, racism, and microaggressions [3]. Findings of elevated risk within student subgroups necessitate greater attention within prevention initiatives to issues of power and identity. Examination of the intersections between oppression and sexual violence is also critically needed [22]. 

Furthermore, the findings present significant implications for the safety and health of students of color and sexual and gender minority students, as research indicates links between experiences of USCV and suicide risk, PTSD, and depression among these subgroups [3, 23]. Transforming existing prevention programming, policies, and practices on campus can help prevent further experiences of victimization, but intervention programming that is culturally responsive, LGBTQ+-affirmative, and trauma-informed is vital for supporting students who have already been victimized. Finally, demographic variation in USCV reinforces the need for alternative response and reporting options for sexual and gender minority and racial/ethnic minority students who have experienced USCV. Qualitative research with student subgroups consistently highlights that minority students may be less likely to utilize traditional campus reporting frameworks such as Title IX due to stigma, fear of being disbelieved, exposure to racist stereotypes, and fear of reflecting poorly on their communities [24]. The current findings provide a window into the differential impact of USCV among student subgroups and can serve as a critical launching point for public health initiatives that take into account unique experiences and barriers to support.

## Data Availability

No datasets were generated or analysed during the current study.
